# Unexpected outcome after expectant management of ectopic pregnancy in two persons

**Published:** 2013-12

**Authors:** Reihaneh Hosseini, Zahra Asgari, Ashraf Moini

**Affiliations:** 1 *Department of Obstetrics and Gynecology, Arash Women’s Hospital, Tehran University of Medical Sciences, Tehran, Iran.*; 2 *Department of Endocrinology and Female Infertility at Royan Institute for Reproductive Biomedicine, ACECR, Tehran, Iran.*

**Keywords:** *Ectopic pregnancy*, *Expectant management*, *Conservative treatment*

## Abstract

**Background:** Ectopic pregnancy is one of the main problems in women in reproductive age that needs special attention and appropriate strategy should be managed. In some cases expectant management seems good strategy without any medicine or surgery and their possible side effects. But are the outcomes always the same? Which outcomes should we expect?

**Case:** We have reported 2 patients whose ectopic pregnancy had been managed conservatively and they had sustained pain for several months which needed surgery to resolve.

**Conclusion:** In the case of ectopic pregnancy, it is important for the clinician to select the patient meticulously and be aware of common and rare consequences of her treatment.

## Introduction

An ectopic pregnancy (EP) is a developing embryo in an area other than endometrial cavity. Although many of patients have the triad of pain, vaginal bleeding and retardation of the menses, this classic feature is absent in more than half of the patients (1). Even with advanced tests and high resolution sonographies, about 40-50% of women with ectopic pregnancy are missed in the first visit (2). 

Also there are some challenges in management strategies. It is believed that for non surgical management, we should have a good risk assessment and the key of success is in appropriate selection (3). In conservative management, we can avoid side effects of methotroxate and surgery but we should first evaluate the short term and long term outcomes of this method. Lui *et al* reported a series of successful conservative management of tubal pregnancy and Hill *et al* has shown two persistent trophoblastic tissues which were treated with metotroxate (4, 5). 

In Cassik *et al* study, they had failure in 5 patients out of 7, due to rupture of tube or pain in the first two weeks in combination with plateau level of hCG (6). Korhonen and van Mello *et al* in their studies reported identical success rate for methotroxate and expectant management (7, 8). The final point of their study was undetectable βhCG which took in average 27 days to get (7). 

In this report we have two patients whose ectopic pregnancies had been managed conservatively (one of them unintentionally) and they suffered from unexpected aftermaths. We could not find the same report in literature searching. Although Atri reported surgical intervention in 5 cases of expectant management despite of declining βhCG because of the pain, that was an acute problem which was found in short term follow up (9). These cases will be useful for clinicians to re-evaluate initial risk factors and to know probable problems when choosing expectant management.

## Case report

**Case 1**

The patient was a 28-year old woman primipara with c/s 7 years ago. She came to our clinic with a history of abnormal uterine bleeding from 2 years before. She was pregnant two years ago and he had experienced an episode of vaginal bleeding in 6 weeks of pregnancy without any previous sonography. So, she was undergone curettage due to diagnosis of incomplete abortion. After that her menses were irregular and she had frequently a sustained annoying pain in her lower abdomen almost always. 

**Figure 1:**
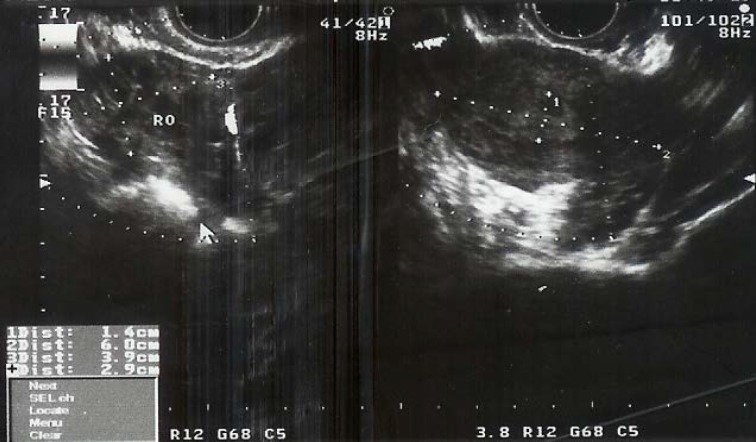
The ultrasound of patient that shows suspicious mass in right adnex (white arrow).

She had several negative pregnancy tests. Her βhCG level was less than 2 and in sonography the endometrial thickness was13 mm with a fine heterogenicity and one local hypoechogenicity with 7×4mm diameter, which was probably endometrial polyp. The left adnex was normal and there was a mix echo lesion in 17×24 mm diameters with an echolucency area around it with 14 mm diameter in right ovary (Figure 1). It had abnormal circulation in color Doppler and was suspicious to aneurism. In physical exam the abdomen was soft without any tenderness or palpable mass although the patient was obese and it had made the exam of her abdomen and adnexes difficult. 

In her MRI report there was an approximately 23×17 mm mass separable from right ovary with T_1_ low and T_2_ high signal. No fat signal is seen in the mass. There was also no evidence of aneurism. The patient was undergone laparoscopy and hysteroscopy due to pain and complex adnexal mass and abnormal uterine bleeding. In laparoscopy the left adnex was normal. In the right adnex it was an old tubal pregnancy that has involved almost half of the tube with significant calcification in the mass but there was not any rupture or hemorrhage evidences in the tube or any adhesion around it. 

The salpangostomy was done and the calcificated mass was extracted completely. In hysteroscopy there was a little polyp and it was extracted. The pathology report of tubal mass was ghost of chorionic villi compatible with degenerated products of conception. Three months later the patient was good with no pain in her lower abdomen. We obtained informed written consent from this patient and the next one to use her data in our report.

**Case 2**

A 42-year old woman primipara with a c/s 9 years ago referred to our center. When she came to our clinic in October, she had suffered from mild pain in her lower abdomen from 2 months ago. She was hospitalized 3 months ago with a positive pregnancy test and vaginal bleeding in another center in order to rule out ectopic pregnancy. At that time she had a 32 mm mixecho mass in left adnex in vaginal sonography suspicious to EP or hemorrhagic cyst and her βhCG serum level had fallen from 693 to 46 IU/L during 10 days. Also, as she had reported cessation of bleeding. So, she was discharged with probable diagnosis of absorbed EP or abortion. 

After 3 months, the patient came to our clinic with sustained lower abdominal pain that was mild but had bothered her. In physical exam the abdomen was soft with mild tenderness in her right lower quadrant. In sonography report the endometrial thickness was 3.5mm and one solid mass in right adnex with 58×34mm diameter. The serum βhCG level was 20 (the threshold of our kit is: positive >25, borderline 10-25 and negative <10) and other tumor markers were normal. We chose laparascopy for patient because of pain and solid mass. 

In laparoscopy there was a tubal pregnancy in right tube that had contained two third of tube and there was organized clots in it without rupture or active hemorrhage. Because of the large size of the EP and complete distortion of tube, salpangectomy was done. After three months the patients was good without any pain.

## Discussion

EP is one of the main problems for women in reproductive age. In past years it was accompanied with high mortality rate but nowadays as a result of modern methods, diagnosis is very earlier and then contemporary treatments are more conservative than in the past. One of those treatments is expectant management. It may end in tubal abortion, spontaneous regression or tubal rupture but the success rate has reported 50-70% (8, 9). Many experts advise follow up until βhCG levels be undetectable. But according to these cases it seems that even a low level of βhCG will not guarantee eradication of the problem. 

Although a threshold value of 200IU/L of βhCG has been suggested and some authors use hCG ratio (hCG 48h/hCG 0h) <1 as indication for expectant management but it seems that other factors such as size of the mass are also important to predict complete absorption of EP (10, 11). Korhonen and van Mello *et al* in their trials consider patients with βhCG levels up to 1500 IU/L for expectant management and their outcomes were similar to methotroxate group (7, 8). Some evidence indicates that the long term outcomes are comparable to those achieved with medical and surgical treatment (13-16) but these examples indicate that the consequences of expectant management and other treatments will not be always the same not only in short term but also in long term. 

Although a problem is that these cases had not been picked up intentionally, and they had not been followed until undetectable βhCG and in first case we didn^’^t have the baseline βhCG, it seems that they had no acute, short term problem. Many of studies have focused on short term outcomes. For example Lui *et al* reported 100% success rate in their expectant management cases, but they considered negative βhCG test as a successful treatment but in our cases and also in some cases of Atri study, the consequences continued even after decline in βhCG level (4, 9). Retained ectopic tissue after conservative management can be biochemically active and be detected by βhCG as Hill *et al* reported in 1990, but this tissue may have other effects even if it is not alive, such as pressure due to adjacent hematoma and it is the probable explanation for our patient’s situation (5). Also, sometimes you can see a decline in βhCG after abortion of ectopic tissue into abdominal cavity followed by bleeding which can lead to surgical intervention. Edozien believes that there is an urgent need for risk management in non-surgical management of EP (17). 

Additionally, the clinician should be aware of probable continuity of problems even after negative βhCG test. Furthermore, some mistakes are made in diagnosis of EP. These mistakes will reduce if meticulous attentions be paid on every person who has symptoms of abortion without any documented evidence of intrauterine pregnancy and it will eradicate some complications in next steps.

## Conclusion

It seems that in addition to a threshold of β-hCG level, some other factors should also be considered for expectant management of EP. It may be possible that precise patient selection beside long term follow up can improve outcomes.
